# Radical prostatectomy for high-risk prostate cancer | *Opinion: YES*

**DOI:** 10.1590/S1677-5538.IBJU.2019.03.02

**Published:** 2019-07-27

**Authors:** Leonardo O. Reis, Rodrigo Montenegro, Quoc-Dien Trinh

**Affiliations:** 1 UroScience, Pontifícia Universidade de Campinas - PUC, Campinas, SP, Brasil; 2 Departamento de Urologia, Universidade Estadual de Campinas - UNICAMP, Campinas, SP, Brasil; 3 Division of Urological Surgery and Center for Surgery and Public Health, Brigham and Women’s Hospital, Harvard Medical School, Boston, MA, USA

**Keywords:** Prostatic Neoplasms, Prostatectomy, Radiotherapy

## INTRODUCTION

Prostate cancer is the commonest non-skin malignancy in men. In most cases, prostate cancer has an indolent course however approximately 30,000 still die from the disease every year. Indeed, a subset of men will present with potentially lethal high-risk prostate cancer at diagnosis. We believe that this proportion will increase as fewer men are screened for prostate cancer, amidst ongoing concerns about overdiagnosis and overtreatment.

The American Urological Association - AUA defines high-risk prostate cancer as PSA ≥20 ng/ml or biopsy Gleason score >7 (ISUP Grade 4/5) or clinical T3. Patients with high-risk prostate cancer have different treatment options including radical prostatectomy (RP), external beam radiotherapy (EBRT) with androgen deprivation therapy (ADT) or EBRT plus brachytherapy (BT) and ADT (1, 2). For many years, EBRT has been the primary treatment option in this population, but the trend has been shifting towards more RP in the most contemporary era. While ideally the evidence should serve as a guide for treatment decisions, the reality of clinical practice is much more complex. Gray et al. showed that sociodemographic factors such as race, insurance and income have a strong influence on treatment choice (3). In parallel, the scientific evidence supporting surgery as part of the initial treatment protocol has been growing in the past decade, even though a sufficiently powered randomized clinical trial is lacking.

Patients are faced with a complex decision-making process when presenting with high-risk prostate cancer. While disadvantages of surgery for high-risk patients include perioperative risks and potentially worse quality of life, urinary and sexual toxicities, especially if patients require adjuvant EBRT+ADT, advantages of surgery for high-risk patients include accurate staging with immediate and excellent local control promoting accurate grading and staging, removal of benign sources of PSA guiding future therapy and possibly avoiding ADT, added to the potential of avoiding urinary (obstructive/irritative) and bowel toxicities and risk of secondary malignancies (4–6).

The comparative effectiveness of different treatment strategies for high-risk prostate cancer is a matter of intense debate. Wallis et al. conducted a meta-analysis of 118,830 patients evaluating the overall mortality and prostate cancer specific between patients treated with RP or radiotherapy. They found that radiotherapy for prostate cancer was associated with an increased risk of overall and prostate cancer-specific mortality compared with RP based on observational data with low to moderate risk of bias (7). In subgroup analyses, they found that the greatest benefit was observed among high-risk prostate cancer patients (HR: 1.83, 95% CI: 1.51-2.22, p=0.0001). Similarly, Petrelli et al., conducted a meta-analysis examining the survival outcomes among patients with only high-risk prostate cancer treated with RP or radiotherapy. They also found better overall and prostate cancer-specific survival for patients treated with surgery compared with radiotherapy. RP is associated with a 44% decreased risk of prostate cancer-specific mortality (HR: 0.56, 95%CI: 0.37-0.85, p=0.007) (8). Interestingly, a recent paper by Ennis et al., relying on data from the National Cancer, showed that there was no difference in overall survival between patients treated with RP versus EBRT plus BT with or without ADT while EBRT plus ADT was associated with lower survival (9). However, another study relying on the same dataset, limited to younger and healthy men (to limit the risk of other cause mortality as this dataset does not identify the cause of death) presenting with high-risk localized prostate cancer, Berg et al. observed that RP was associated with a statistically significant overall survival benefit compared to EBRT plus BT (10) (HR (EBRT plus BT compared to RP): 1.22, 95% CI: 1.05-1.43). However, it is still important to acknowledge, as surgeons, that patients treated with RT are very different from those treated with surgery and residual confounding cannot be excluded. Moreover, most studies are biased by retrospective design or underpowered to draw any conclusion on the efficacy of the treatments (11–14), which also undermine the conclusions of the available meta-analyses, limited to observational studies (7, 8).

A key benefit of surgery is to provide more accurate staging, as well as prognostic information (15). A problem of broad all-encompassing diagnostic categories is that some patients have much more aggressive disease than others, even within the same risk stratification group. A patient presenting with a single core of Gleason 4+4 prostate cancer likely has a different prognosis than a patient presenting with Gleason 5+5 prostate cancer in all cores invading both seminal vesicles on imaging. Specifically, a study showed that 60% of patients with a biopsy Gleason score 8 was downgraded following radical prostatectomy (1). Another concern is that many cases that improvements in imaging techniques such as the MRI has led to a higher proportion of men diagnosed with clinical stage T3 disease - while these patients are obviously at a higher risk of recurrence and/or need for adjuvant EBRT+ADT, it would be unfair to say that the prognosis of such patients is the same as clinical T3 disease found on the basis of a digital rectal exam. For such cases, and many others, RP offers the hope of cure with surgery alone, potentially avoiding the toxicities of prolonged ADT, which for many, are more severe than toxicities of RT. Indeed, ADT is associated with a plethora of quality of life affecting, and potentially life-threatening side-effects such as: metabolic syndrome, cardiovascular toxicities (16), cognitive dysfunction (17), liver disease (18), osteoporosis, etc. Even among those who are not cured with surgery alone, the duration of adjuvant ADT (given with EBRT) in the setting of adverse features at RP is manifold shorter than the ADT accompanying EBRT as a first-line treatment for high-risk prostate cancer.

## CONCLUSIONS

While we fully acknowledge the limitations of the retrospective data cited in the current review (19), we believe that there is more than enough data to support RP as a first-line treatment for high-risk prostate cancer in the right surgical candidate. Given the lack of level 1 evidence, it is important to discuss the pros and cons of each treatment option (RP vs EBRT+BT+ADT vs. EBRT+ADT) in a multidisciplinary setting. It is also important to acknowledge that novel treatment approaches centered around surgery are being tested as we speak - for example the use of neoadjuvant androgen deprivation therapy prior to RP for high-risk prostate cancer, using next generation molecules, is the subject of several ongoing trials (20–23).
